# Preparedness of outpatient health facilities for ambulatory treatment with all-oral short DR-TB treatment regimens in Zhytomyr, Ukraine: a cross-sectional study

**DOI:** 10.1186/s12913-020-05735-z

**Published:** 2020-09-21

**Authors:** Tinne Gils, Chinmay Laxmeshwar, Marve Duka, Khachatur Malakyan, Olga Valentinovna Siomak, Vitaly Stephanovich Didik, Natalia Lytvynenko, Yana Terleeva, Dmytri Donchuk, Petros Isaakidis

**Affiliations:** 1 Médecins Sans Frontières, 64 Berdychivska St., Huiva, Zhytomyr, Oblast Ukraine; 2 Zhytomyr Regional Tuberculosis Dispensary, Zhytomyr, Ukraine; 3 National Institute of Phthisiology and Pulmonology named after F.G. Yanovsky of National Academy of Medical Sciences of Ukraine, Kyiv, Ukraine; 4 Department of Tuberculosis Programme Coordination, Public Health Centre of the Ministry of Health of Ukraine, Kyiv, Ukraine; 5 Southern Africa Medical Unit, Médecins Sans Frontières, Cape Town, South Africa

**Keywords:** DR-TB, Patient-centred care, Outpatient care, Anti-microbial resistance, Quality of care, DR-TB health system

## Abstract

**Background:**

Ukraine has a high burden of drug-resistant tuberculosis (DR-TB). Mental health problems, including alcohol use disorder, are common co-morbidities. One in five DR-TB patients has human immunodeficiency virus (HIV). As part of health reform, the country is moving from inpatient care to ambulatory primary care for tuberculosis (TB). In Zhytomyr oblast, Médecins Sans Frontières (MSF) is supporting care for DR-TB patients on all-oral short DR-TB regimens. This study describes the preparedness of ambulatory care facilities in Zhytomyr oblast, Ukraine, to provide good quality ambulatory care.

**Methods:**

This is a retrospective analysis of routinely collected programme data. Before discharge of every patient from the hospital, MSF teams assess services available at outpatient facilities using a standardised questionnaire. The assessment evaluates access, human resources, availability of medicines, infection control measures, laboratory and diagnostic services, and psychosocial support.

**Results:**

We visited 68 outpatient facilities in 22 districts between June 2018 and September 2019. Twenty-seven health posts, 24 TB-units, 13 ambulatories, two family doctors and one polyclinic, serving 30% of DR-TB patients in the oblast by September 2019, were included. All facilities provided directly observed treatment, but only seven (10%) provided weekend-services. All facilities had at least one medical staff member, but TB-training was insufficient and mostly limited to TB-doctors. TB-treatment and adequate storage space were available in all facilities, but only five (8%) had ancillary medicines. HIV-positive patients had to visit a separate facility to access HIV-care. Personal protective equipment was unavailable in 32 (55%) facilities. Basic laboratory services were available in TB-units, but only four (17%) performed audiometry. Only ten (42%) TB-units had psychosocial support available, and nine (38%) offered psychiatric support.

**Conclusion:**

Outpatient facilities in Zhytomyr oblast are not yet prepared to provide comprehensive care for DR-TB patients. Capacity of all facilities needs strengthening with trainings, infection control measures and infrastructure. Integration of psychosocial services, treatment of co-morbidities and adverse events at the same facility are essential for successful decentralisation. The health reform is an opportunity to establish quality, patient-centred care.

## Background

Anti-microbial resistance is among the most important public health concerns globally and tuberculosis (TB), the world’s leading cause of death by an infectious disease, contributes to this threat [1, 2]. In 2018, only one third of estimated patients with drug-resistant tuberculosis (DR-TB) were initiated on second-line regimens, and only 56% among them were treated successfully [2]. Despite gaps in strategies to treat and retain patients, limited studies examine the quality of routine DR-TB care, including access to patient-centred services, quality of treatment monitoring, follow-up of adverse events and psychosocial services [3]. There is ample evidence supporting community-based ambulatory DR-TB care over prolonged hospitalisation, with ambulatory care showing better treatment outcomes, more patient satisfaction and a lower cost [4–8]. All-oral regimens including bedaquiline (BDQ) and delamanid (DLM) allow for easier decentralisation compared to injectable-containing regimens, provided adequate monitoring of adverse events is ensured [9]. The World Health Organization (WHO) proposed a checklist with minimum requirements to assess implementation sites of new TB-drugs on capacity to provide good quality of care [10].

Ukraine is a country with one of the highest DR-TB burdens world-wide, with 24 and 58% of patients diagnosed with DR-TB among new and previously treated TB-cases, respectively [11]. There were an estimated 13,000 new DR-TB cases but only 7926 initiations in 2018. Treatment success among cases initiated in 2016 was 49% [2]. Human immunodeficiency virus (HIV) co-infection among TB-patients in Ukraine was 23% in 2018 [2, 12]. TB-related stigma is more common in HIV-positive patients, patients suffering from alcohol use disorder or intravenous drug-use and prisoners [11, 13]. The Ukrainian national TB-programme has historically been a vertical programme with centralised TB-care, daily directly observed treatment (DOT) and months to years of hospitalisation for TB-patients. Until 2019, DR-TB treatment was only provided at central TB-dispensaries in the oblasts [11].

A Ukrainian health systems reform foresees decentralisation of services which will put peripheral family doctors in charge of a patients’ health, including detection, diagnosis and treatment for TB and co-morbidities from 2020 [13, 14]. Patients will be able to choose their physician, who is expected to comply with international standards of care [14]. BDQ and DLM were registered in Ukraine by early 2019 and their use following WHO treatment protocols is endorsed [9, 15, 16]. However, new drugs are not yet widely available and clinicians commonly prescribe long-term regimens including injectable drugs for DR-TB [17].

Médecins Sans Frontières (MSF) has been working in partnership with the regional TB-Dispensary of Zhytomyr Regional Council, the National TB-programme of the Ministry of Health of Ukraine, and the F.G. Yanovsky NAMSU National Institute of Phthisiology and Pulmonology in Zhytomyr oblast since 2018, and started a quasi-experimental trial with an all-oral short regimen including BDQ and DLM to treat DR-TB in March 2019. Before discharge of patients from the hospital, MSF teams assess outpatient facilities on availability of essential services, as proposed by the WHO [10]. This assessment was not embedded in the trial protocol and was designed to provide additional information on the quality of care available in outpatient facilities. To our knowledge, no study has presented a systematic assessment of preparedness of ambulatory DR-TB services in this setting. As the country-wide decentralisation of TB-services and all-oral DR-TB treatment roll-out starts, we aimed to discuss the readiness of outpatient facilities in Zhytomyr oblast, Ukraine, for quality decentralised care for DR-TB patients.

## Methods

### Study setting

Zhytomyr oblast is situated in central Ukraine. An oblast is the administrative equivalent of a province. Zhytomyr oblast had a population of around 1,25 million in 2017, spread over 24 districts, including the capital district where the regional TB-dispensary is situated. The oblast had an estimated TB-incidence of 76.5 and a TB-prevalence of 96.8 per 100,000 people in 2018. In 2018, 21% (269) of registered TB-cases had DR-TB (13). In November 2019, facilities providing ambulatory TB-care in Zhytomyr oblast included 433 medical midwife/paramedic points and feldsher points (hereafter called health posts), 234 ambulatories, 57 primary health care centres (PHC, including 11 polyclinics), and 25 TB-units. There were 795 family doctors in the oblast [18]. Treatment for DR-TB in Zhytomyr oblast is initiated at and coordinated through the regional TB-dispensary, a tertiary level facility, in Zhytomyr city. MSF is augmenting counselling services and reducing the duration of hospitalisation for DR-TB patients by providing support for active follow-up of patients after discharge [19]. Discharge from the regional TB-dispensary is currently possible after smear-conversion and a positive medical advice, while considering patient preference. All patients who are referred to outpatient facilities other than a TB-unit, visit the nearest affiliated TB-unit once a month for clinical assessment by a TB-specialist, laboratory tests, side effect monitoring and their management.

### Study design and population

This retrospective analysis uses routinely collected cross-sectional data to evaluate available services and their quality in outpatient facilities for DR-TB in Zhytomyr oblast, Ukraine. All facilities to which at least one DR-TB patient was discharged between June 2018 and September 2019, after initiation at the TB-hospital, were included. Location of discharge was influenced by proximity to the patients’ home and personal choice. Facilities which were closed during the visit period were excluded.

### Variables and definitions

Table 1 presents the definitions used for the types of facilities to which patients were discharged.

**Table 1:** Definitions used for outpatient health facilities in Ukraine

Facility type	Definition
**Ambulatory**	A subunit of the treatment and preventive services of the primary health care centre (PHC), providing primary care to the population of its catchment area, including emergency medical care. An ambulatory can serve over 1500 people in the city and over 1200 people in rural areas, and at least one family doctor works there [20].
**Family doctor** or general practitioner	A doctor who has received specialised multidisciplinary training to provide primary health care to patients of any age and gender. Family doctors can work independently or in an outpatient clinic [20].
**Health post** or medical midwife/paramedic point or feldsher point	A separate structural unit linked to the ambulatory arm of the PHC centre, which provides pre-hospital medical care to the population in one or more localities where there are no other providers of free primary care. A health post is administered by a clinician (called feldsher, hereafter referred to as nurse practitioner) to whom residents of the locality(s) where the health post is located are attached [20].
**Polyclinic**	An outpatient unit of a multidisciplinary or specialised hospital (secondary level) where consultations by different medical specialist are available. Prescriptions are usually filled at a pharmacy outside the polyclinic. The number of doctors and nurses available is proportional to the population of the area served [21].
**Primary health care centre**	A health care facility established to meet the needs of the population in the catchment area in primary care. The structure of the PHC centre includes a treatment and prevention department (i.e. ambulatories and health posts) and an administrative unit [20].
**TB-unit** or TB-cabinet	A specialised institution where preventive, diagnostic and treatment services for tuberculosis are provided. Such institutions may be a structural subdivision of a hospital, or outpatient facilities [22].

The names used in this text are written in bold

*PHC* primary health care centre, *TB* tuberculosis

Variables collected for all facilities were related to access, presence and training of human resources, availability of medicines and related amenities, and presence of infection control measures. In TB-units we also assessed availability of laboratory, diagnostic and psychosocial services. WHO definitions were used for DR-TB treatment and care-related terms [9]. Social support was defined as provision of patient-specific services including food packages, hygiene kits, home visits, transport vouchers, firewood, or monetary support for treatment of chronic co-morbidities e.g. diabetes, hypertension. Counselling was defined as individual or group-sessions performed by a nurse, social worker or psychologist to address issues related to adherence and quality of life. Psychiatric services where defined as access to a psychiatrist. Ancillary medicines were defined as any medicines to treat adverse events of DR-TB treatment, including antihistamines, anti-inflammatory, and anti-emetic medicines. Haematology tests included haemoglobin measurement, white and red blood cell count, differential leucocyte count and erythrocyte sedimentation rate. Biochemistry and electrolyte testing included creatinine, liver enzymes, potassium, magnesium and chloride measurement. If all of these were available, the service was considered present.

### Data collection, verification, and analysis

Facility assessment data were collected by trained health workers. Two questionnaires were developed based on WHO guidance for implementation of new TB-regimens; one for TB-units and one for other outpatient facilities [10]. A Ukrainian version of the questionnaire was piloted through facility visits, accompanied by an epidemiologist, and adapted. Questionnaires were translated to English and back translated to Ukrainian by a second translator. Whenever possible, availability of services was verified physically, however, we relied on staff response for variables related to access and psychosocial services. Completed forms were reviewed by an epidemiologist, and missing data were completed during subsequent visits or facilities were contacted by telephone. Data were entered in Microsoft Excel and imported into Stata *(Version 15.0, StataCorp)* for analysis. Frequencies and percentages were used for description of categorical variables and medians with interquartile ranges for continuous variables.

## Results

Between June 2018 and September 2019, 68 outpatient facilities in 22 (92%) districts were visited: 13 ambulatories, two family doctors, 28 health posts, a polyclinic and 24 TB-units (one paediatric). One temporarily closed health post (two nurses were on maternity leave and the patient was linked to another facility) was excluded from analysis. The results of the assessment are shown in Table 2.

**Table 2:** Outcomes of assessed variables of outpatient facilities, June 2018–September 2019, Zhytomyr oblast, Ukraine

Variables collected	All facilities	Type of facility				
		TB-units	Health posts	Ambulatories	Family doctors	Polyclinic
	(*n* = 67)	(*n* = 24)	(*n* = 27)	(*n* = 13)	(n = 2)	(*n* = 1)
**Access**						
DOT services available (n, %)	67 (100%)	24 (100%)	27 (100%)	13 (100%)	2 (100%)	1 (100%)
Weekday working hours (median, IQR) (*n* = 65)	8 (7–9)	8 (7–8)	8 (7–9)	8 (7–9)	7, 9	6
Open after 5 pm (n = 65) (n, %)	10 (15%)	4 (17%)	3 (12%)	2 (15%)	1 (50%)	0
Open seven days per week (n, %)	7 (10%)	0	2 (7%)	4 (31%)	0	1 (100%)
Distance in kilometres from TB-unit (*n* = 34)	15 (10–20)	na	15 (8–20)	19 (11–24)	6, 10	─
Public transport available to reach TB-unit (*n* = 39) (n, %)	37 (95%)	na	22 (96%)	13 (100%)	2 (100%)	─
**Clinical human resources**						
≥ 1 doctor available (n, %)	44 (66%)	23 (96%)	7 (26%)	12 (92%)	2 (100%)	0
≥ 1 nurse practitioner available (n, %)	24 (36%)	0	17 (63%)	6 (46%)	1 (50%)	0
≥ 1 nurse available (n, %)	61 (91%)	24 (100%)	21 (78%)	13 (100%)	2 (100%)	1 (100%)
Total number available per facility (median, IQR)	3 (2–5)	3 (2–5)	2 (1–2)	6 (4–10)	3, 5	3
≥ 1 staff with ≥ TB-training in last 2 years (*n* = 55) (n, %)	32 (58%)	21 (91%)	6 (33%)	5 (42%)	0	─
**Presence of medicines and related amenities (n, %)**						
TB-medicines	67 (100%)	24 (100%)	27 (100%)	13 (100%)	2 (100%)	1 (100%)
Ancillary medicines (*n* = 61)	22 (36%)	5 (21%)	12 (55%)	5 (42%)	0	0
Of which provided by MSF (*n* = 22)	17 (77%)	5 (100%)	10 (83%)	2 (40%)	na	na
Antiretroviral medicines	0	0	0	0	0	0
Storage space	67 (100%)	24 (100%)	27 (100%)	13 (100%)	2 (100%)	1 (100%)
Regular supply system (*n* = 62)	62 (100%)	24 (100%)	23 (100%)	13 (100%)	2 (100%)	─
Drinking water (*n* = 64)	40 (63%)	10 (42%)	18 (72%)	11 (85%)	1 (50%)	─
**Presence of infection control measures (n, %)**						
Functional ultra-violet light (*n* = 58)	44 (76%)	22 (92%)	10 (45%)	11 (92%)	2 (100%)	─
≥ 1 respirator (n = 58)	26 (45%)	20 (83%)	3 (15%)	2 (16%)	1 (50%)	─

─ indicates missing data, na = not applicable for this type of facility

*DOT* directly observed treatment, *HIV* human immunodeficiency virus, *IQR* interquartile range, *MSF* Médecins Sans Frontières, *TB* tuberculosis

The facilities included represented 6% of ambulatories, < 1% of family doctors, 6% of health posts, 9% of polyclinics (or 2% among PHC), and 96% of TB-units in Zhytomyr oblast. Seventy patients on BDQ/DLM-containing regimens were followed in these facilities by the end of September 2019, accounting for 51% (70/138) of DR-TB patients on BDQ and 30% (70/236) of DR-TB patients on treatment in Zhytomyr oblast.

### Human resources

**Fig. 1:**
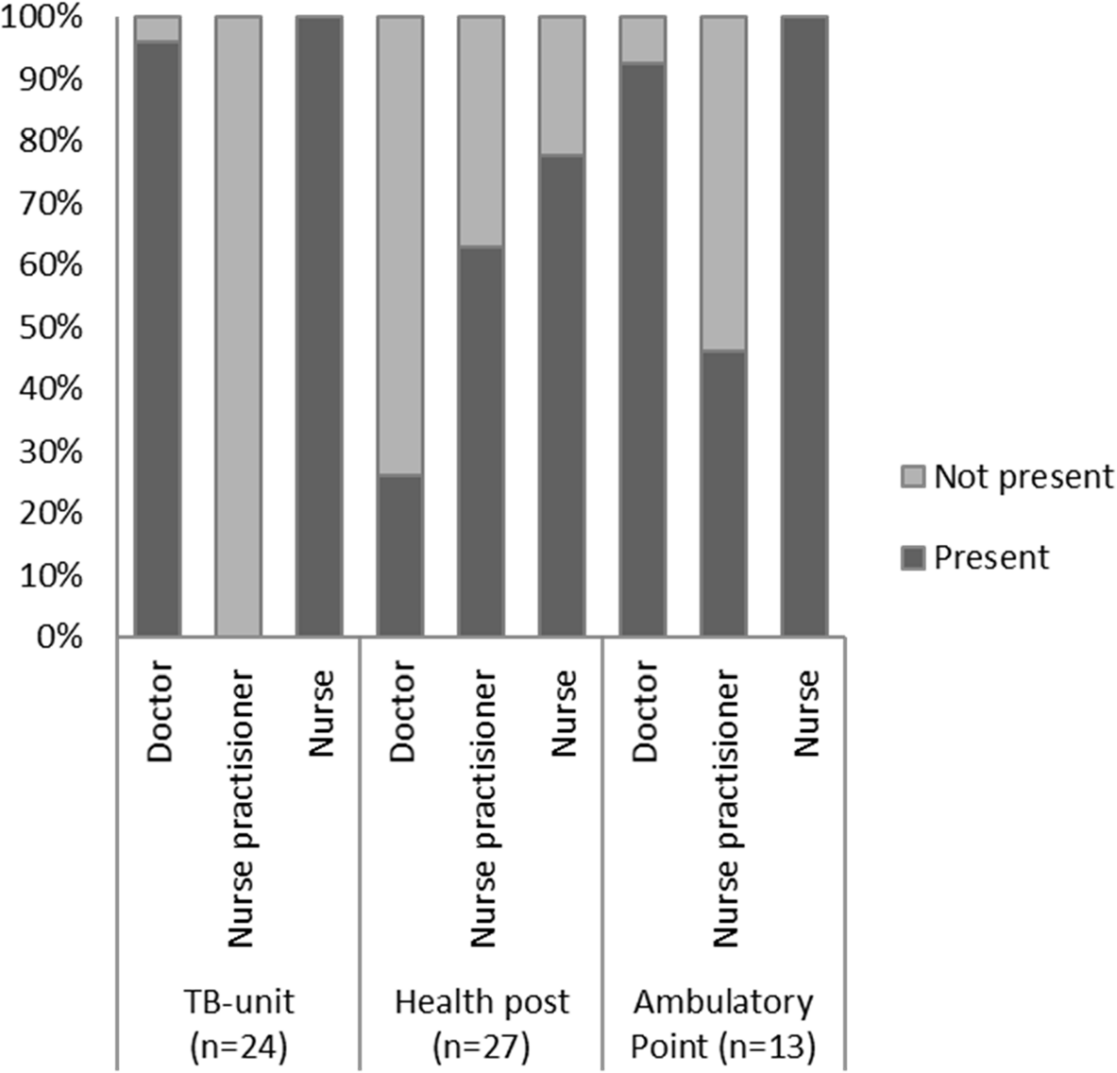
Proportion of outpatient facilities with clinical staff present, June 2018–September 2019, Zhytomyr oblast, Ukraine. TB = tuberculosis

All facilities had at least one doctor, nurse practitioner or nurse available (Fig. 1). Only seven 26%) health posts had a doctor, and in four (15%) they worked part-time. TB-training content was mostly focussed on management of drug-susceptible TB.

### Medicines and related amenities

All facilities stored TB-medicines in a dedicated storage space (either a room or a cabinet). All facilities reported having a regular supply system in place, but two TB-units reported having had one and five stock-outs of TB-medicines in the last year. None of the visited facilities stored anti-retroviral medicines (ART). To receive HIV-care, patients had to visit one of five ART-centres in the oblast monthly. In facilities where no drinking water was available (24, 38%) patients brought their own.

### Laboratory and diagnostic services in TB-units

**Fig. 2:**
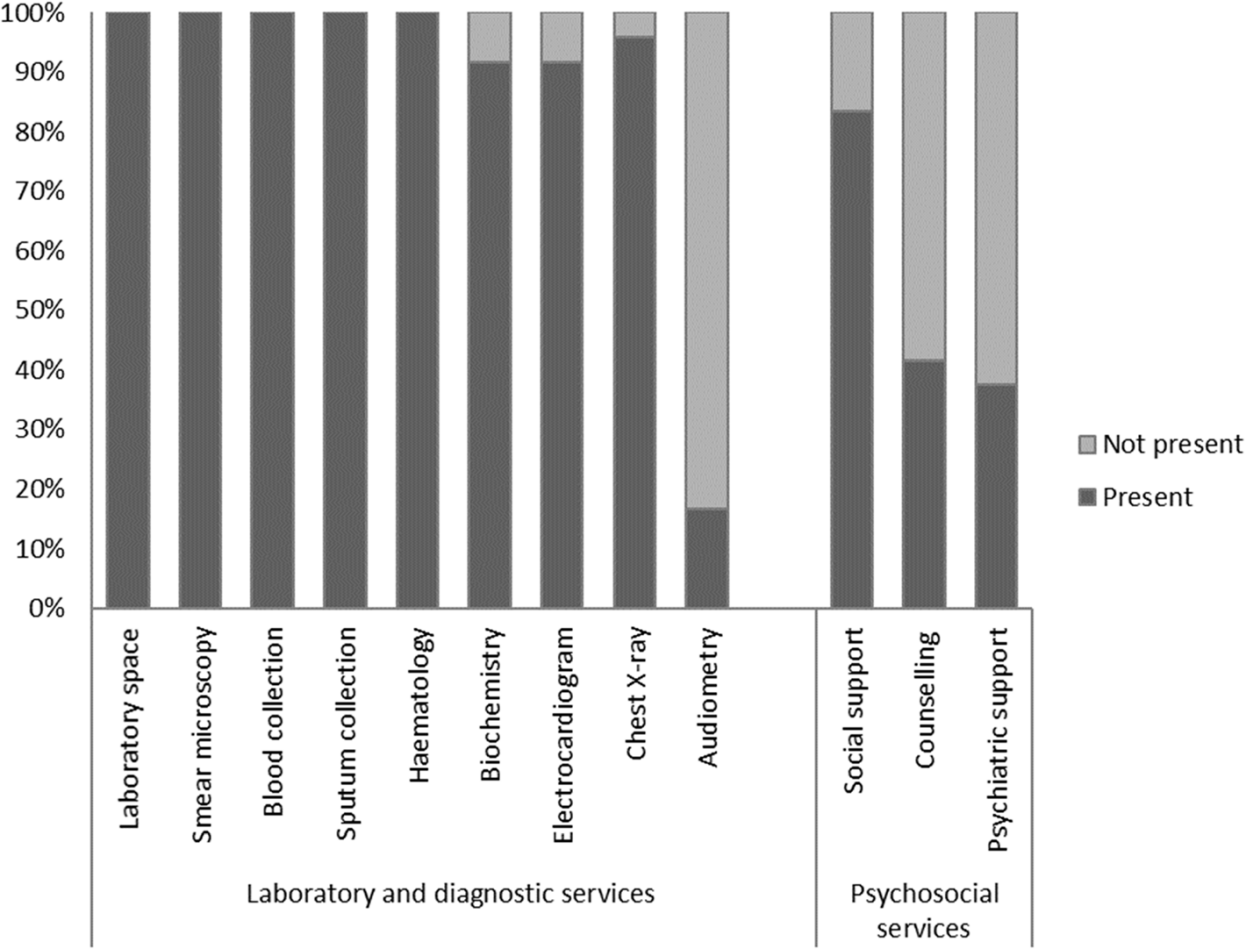
Proportion of TB-units with laboratory, diagnostic and psychosocial services available, June 2018–September 2019, Zhytomyr oblast, Ukraine. TB = tuberculosis

All 24 (100%) TB-units had a laboratory space and performed sputum collection, blood collection and haematology. Chest X-ray was available at 23 (96%) TB-units, 22 (92%) had biochemistry and electrocardiogram, and four (17%) performed audiometry (Fig. 2).

### Psychosocial services in TB-units

Twenty (83%) TB-units reported providing social support services, ten (42%) counselling services, and nine (38%) psychiatric support. When social support or counselling was available, external actors provided the service in 15 (75%) and eight (80%) instances, respectively.

## Discussion

This is the first study assessing availability of services for ambulatory DR-TB treatment with all-oral short regimens in Ukraine. Our data show that ambulatory facilities in Zhytomyr are not yet fully prepared to provide comprehensive treatment and care for DR-TB patients. Although basic services were present in most facilities, essential elements for patient-centred care, such as psychosocial support and integrated treatment for co-morbidities and adverse events, were found lacking.

Access to facilities was mostly possible by public transport but most facilities were not open after regular office hours. DR-TB treatment is known to lead to catastrophic costs for patients and their households, including travel-costs and due to time spent at the facility for DOT [23, 24]. Instead of enforcing DOT, patients-specific approaches on where, when and how to access treatment should be sought [25].

All facilities had medical staff available. TB-training was limited, especially in non-TB-units, where TB-doctors were less present. Training increases knowledge on TB and is likely to reduce stigma [26, 27]. TB-patients in Ukraine reported in interviews that they experience stigmatising behaviour more commonly from non-specialised and junior health care workers [28]. Continuous education, including specific training on critical changes, such as introduction of new TB-regimens, should be available to all TB-care providers [10]. Staff in newly designated outpatient DR-TB facilities should receive DR-TB training, especially non-TB-specialists in health posts and ambulatories.

Although TB-medicines were present and storage space was acceptable, ancillary medicines were rarely available outside of MSF support, even in TB-units. Stock of minimal ancillary medicines should include drugs to treat common adverse events such as gastro-intestinal disturbances, joint pains, dizziness and skin rashes [29]. These adverse events can be debilitating if left untreated and contribute significantly to a loss of quality of life [30, 31]. ART was unavailable, and HIV-patients had to travel to an ART-centre monthly. One in five TB-patients in Ukraine is HIV-positive, yet there is lack of integration of these services [2]. Integration of HIV/TB-services is effective and cost-effective, and specific WHO-guidance exists for patient-centred TB-services in Eastern Europe and Central Asia [32, 33]. TB-units could be the sites for referral for adverse events management and ART-refills, and they should be adequately supplied with medicines.

Basic infection control measures were unavailable in many facilities, especially health posts, but also in TB-units. Currently patients are only referred to ambulatory care once they become smear negative and are thus non-infectious. The next step in the decentralisation process is the TB-treatment initiation in ambulatory facilities. Basic measures such as ventilation, UV-equipment, and personal protective equipment will be necessary to protect other patients and health-care workers [10].

Laboratory and diagnostic services were available in most TB-units, but audiometry equipment was unavailable. Progressive hearing loss is an irreversible side-effect of injectables, which can be detected and limited through audiometry monitoring [34]. While BDQ is included in national DR-TB protocols in Ukraine, audiometry remains crucial for patients already on injectable-containing regimens [16]. Among potential side-effects of new drugs requiring regular monitoring is the prolongation of the QT-interval [35, 36]. Early safety results indicate a limited risk of cardiac toxicity from BDQ and DLM, and regular electrocardiogram monitoring might become less relevant in the future [37].

Psychosocial services were inconsistently available and if present, provided by external non-governmental actors. A review across 20 countries found pooled prevalence of 25% depression, 24% anxiety and 10% psychosis in DR-TB patients, with stigma, isolation, discrimination and lack of social support as main stressors [38]. In Ukraine, high prevalence of HIV and substance use disorders in DR-TB patients contribute to additional stigma and mental stress [11, 39]. A Ukrainian study found that patient receiving social support are less likely to be lost to follow up [40]. A health service assessment in four central European countries, concluded that tailored, patient-centred services where paramount for good DR-TB care [41].

Availability of ancillary medicines, psychosocial assessment, and essential ART-monitoring services like viral load at implementation sites are minimum conditions for roll-out of new DR-TB treatment according to WHO [3]. Absence of these services hampers delivery of quality DR-TB care [3, 10]. Some benefits of TB-decentralisation are undone if patients need to travel separately to access ancillary medicines, ART or electrocardiography. Integration of services is equally necessary for diabetes, viral hepatitis, substance-use disorder and other mental health conditions [32]. Chronic co-morbidities, as well as permanent disability from side-effects, increase the DR-TB burden and impact patients’ well-being during and after the end of treatment [30, 42]. To limit the impact of these factors in reducing quality of life after treatment, patient-centred interventions should be established during treatment [42]. Roll-out of oral regimens is an opportunity to shift from controlling towards supporting patients to improve adherence with individualised approaches, considering patient reality.

While family doctors are set to become the main healthcare providers for patients under the healthcare reform, only two family doctors were included in this analysis. Non-TB specialists could be reluctant to recruit DR-TB patients due to TB-related stigma, which is common in Ukraine and associated with limited TB-knowledge [43, 44]. In Zhytomyr, so far only two thirds of the population have been linked to family doctors, which is in line with national numbers [18]. A possible explanation is the fact that no clear mechanisms to link patients and doctors have been identified [14, 18]. Such mechanisms for TB, and intensive TB-training for family doctors will be necessary to provide adequate care [14].

Our study has several limitations. We did not assess a representative sample of ambulatory facilities in Zhytomyr, but visited facilities based on the needs of patients being discharged from the regional TB-dispensary. However, these facilities provided care for 30% of DR-TB patients in Zhytomyr, including all outpatients on BDQ/DLM-containing treatment. We used staff responses for results we could not physically verify, which could have led to overestimation of quality of services. For instance, staff might have overstated availability to provide weekend DOT-services. Recall bias might lead to an overestimation of TB-training received in the last two years. The cross-sectional design of our study did not allow tracking improvements over time, and repetition of similar surveys is necessary to evaluate progress.

As decentralisation of TB-treatment and country-wide roll-out of all-oral short regimens is imminent in Ukraine, defining needs and strengthening of ambulatory facilities is essential to ensure that quality care can be provided to DR-TB patients. In the short term, infection control equipment and ancillary medicines should be quantified and supplied to each ambulatory site. Training should be provided on integrated TB-care, including infection control, especially for staff in health posts, ambulatories and family doctors. TB-units in oblast like Zhytomyr could serve as pilot-sites for decentralisation of integrated HIV and TB-services, while including minimum standards for provision of psychosocial and psychiatric services. Similar analyses to ours could be repeated in other oblasts, should include family doctors, and identified issues should be acted upon by dedicated taskforces. A recently developed “Quality of tuberculosis services assessment tool”, including facility, provider and patient assessments could be used for more in-debt assessments in outpatient facilities [45].

The national TB-programme should take advantage of the health reform coinciding with all-oral DR-TB regimen roll-out to provide an integrated training package to healthcare workers, and set ambitious goals for quality DR-TB care for the country. TB patients as end-users could be engaged to monitor quality of services and given the tools to safely report gaps [46].

## Conclusions

Outpatient facilities in Zhytomyr oblast are not well prepared to provide quality services to DR-TB patients on new shorter regimens and need strengthening to provide comprehensive care. Minimum standards should be set for psychosocial support services, adverse events treatment and integrated care and treatment of co-morbidities. The ongoing health reform is an opportunity to establish good quality, patient-centred DR-TB care.

## Data Availability

MSF has a managed access system for data sharing that respects MSF’s legal and ethical obligations to its patients to collect, manage and protect their data responsibility. Ethical risks include, but are not limited to, the nature of MSF operations and target populations being such that data collected are often highly sensitive. Data are available on request in accordance with MSF’s data sharing policy (available at: http://fieldresearch.msf.org/msf/handle/10144/306501). Requests for access to data should be made to data.sharing@msf.org.

## Abbreviations

ART: Anti-retroviral Treatment
BDQ: Bedaquiline
DLM: Delamanid
DR-TB: Drug-Resistant Tuberculosis
DOT: Directly Observed Treatment
HIV: Human Immunodeficiency Virus
IQR: Interquartile Range
MSF: Médecins Sans Frontières
PHC: Primary Health Care Centre
SAT: Self-Administered Treatment
TB: Tuberculosis
WHO: World Health Organization

## References

[1] World Health Organisation. Global Action Plan on Antimicrobial Resistance. Geneva; 2015. https://apps.who.int/iris/bitstream/handle/10665/193736/9789241509763_eng.pdf?sequence=1. Accessed 19 August 2020.

[2] World Health Organization. Global Tuberculosis Report 2019. Geneva; 2019. https://apps.who.int/iris/bitstream/handle/10665/329368/9789241565714-eng.pdf?ua=1. Accessed 19 August 2020.

[3] Udwadia Z, Furin J (2019). Quality of drug-resistant tuberculosis care: gaps and solutions. J Clin Tuberc Other Mycobact Dis.

[4] Ho J, Byrne AL, Linh NN, Jaramillo E, Fox GJ (2017). Decentralized care for multidrug-resistant tuberculosis: a systematic review and meta-analysis. Bull World Health Organ.

[5] Daru P, Matji R, Almossawi J, Chakraborty K (2018). Decentralized , Community-Based Treatment for Drug-Resistant Tuberculosis: Bangladesh Program Experience. Glob Heal Sci Pract.

[6] Zhang H, Ehiri J, Yang H, Tang S, Li Y. Impact of community-based DOT on tuberculosis treatment outcomes: A systematic review and meta-analysis. PLoS One. 2016;11(2):1–19. 10.1371/journal.pone.0147744 PMC4744041 26849656

[7] Cox H, Ford N (2013). Decentralisation of multidrug-resistant-tuberculosis care and management. Lancet Infect Dis.

[8] Bassili A, Fitzpatrick C, Qadeer E, Fatima R, Floyd K, Jaramillo E (2013). Review article: a systematic review of the effectiveness of hospital and ambulatory-based management of multidrug-resistant tuberculosis. Am J Trop Med Hyg.

[9] World Health Organization. WHO consolidated guidelines on drug-resistant tuberculosis treatment. Geneva; 2019. https://apps.who.int/iris/bitstream/handle/10665/311389/9789241550529-eng.pdf?ua=1. Accessed 19 August 2020. 30946559

[10] World Health Organization. Policy Implementation Package for new TB drug introduction. Geneva; 2014. Available from: https://www.who.int/tb/PIPnewTBdrugs.pdf. Accessed 19 August 2020.

[11] Pavlenko E, Barbova A, Hovhannesyan A, Tsenilova Z, Slavuckij A, Shcherbak-Verlan B (2018). Alarming levels of multidrug-resistant tuberculosis in Ukraine: results from the first national survey. Int J Tuberc Lung Dis..

[12] Joint United Nations program on AIDS/HIV. UNAIDS Data 2019. Geneva; 2019. https://www.unaids.org/sites/default/files/media_asset/2019-UNAIDS-data_en.pdf. Accessed 19 August 2020.

[13] Centre for Public Health. Centre for Health Statistics. Ministry of Health Ukraine. Tuberculosis in Ukraine. Kyiv; 2019. https://phc.org.ua/. Accessed 18 Sept 2020.

[14] Romaniuk P, Semigina T (2018). Ukrainian health care system and its chances for successful transition from soviet legacies. Glob Health.

[15] Ministry of Health Ukraine (2016). Order No. 1422 of 29.12.2016. Amendments to the Order of the Ministry of Health of Ukraine of September 28, 2012 No. 751.

[16] Ministry of Health Ukraine. TB Guidelines. Center for Public Health. Kyiv; 2019. Available from: https://phc.org.ua/kontrol-zakhvoryuvan/tuberkuloz/kerivni-dokumenti-z-tb. Accessed 19 August 2020.

[17] Ministry of Health Ukraine (2014). Order No. 620 of 04.09.2014. Uniform clinical protocol of primary, secondary (specialized) and third (highly specialized) health care for adult Tuberculosis.

[18] Ministry of Health Ukraine. E-map of places in the provision of primary health care. Kyiv; 2020. https://nszu.gov.ua/en/e-data/dashboard/elektronna-karta-misc-nadannya-pervinnoyi-mediko-sanitarnoyi. Accessed 19 August 2020.

[19] Médecins Sans Frontières Ukraine. A long and painful journey. Kyiv; 2019. https://msf.exposure.co/a-long-painful-journey. Accessed 19 August 2020.

[20] Ministry of Health Ukraine (2016). Order No. 801 of 29.07.2016. Approval of the Regulations on the Center for Primary Medical (Health) and the provisions on its subdivisions.

[21] Ministry of Health Ukraine (2011). Order No. 1008 of 30.12.2011. Approval of approximate provisions about health care facilities.

[22] Ministry of Health Ukraine (2009). Order No. 514 of 16.07.2009. Approval of the List of Tuberculosis Treatment Facilities.

[23] Prasanna T, Jeyashree K, Chinnakali P, Bahurupi Y, Vasudevan K, Das M (2018). Catastrophic costs of tuberculosis care: a mixed methods study from Puducherry, India. Glob Health Action.

[24] Ramma L, Cox H, Wilkinson L, Foster N, Cunnama L, Vassall A (2015). Patients’ costs associated with seeking and accessing treatment for drug-resistant tuberculosis in South Africa. Int J Tuberc Lung Dis..

[25] Khachadourian V, Truzyan N, Harutyunyan A, Thompson ME, Harutyunyan T, Petrosyan V (2015). People-centered tuberculosis care versus standard directly observed therapy: study protocol for a cluster randomized controlled trial. Trials..

[26] Wu S, Roychowdhury I, Khan M (2017). Evaluating the impact of healthcare provider training to improve tuberculosis management: a systematic review of methods and outcome indicators used. Int J Infect Dis.

[27] Sommerland N, Wouters E, Mitchell EMH, Ngicho M, Redwood L, Masquillier C (2017). Evidence-based interventions to reduce tuberculosis stigma: a systematic review. Int J Tuberc Lung Dis.

[28] Akhmetov’s foundation for Development of Ukraine. Level of knowledge, attitude, practice and behavior of the population of Ukraine and separate social groups on tuberculosis as of 2011. Kyiv; 2013. https://akhmetovfoundation.org/ru/news/fond-rinata-ahmetova-rozvytok-ukrainy-oprylyudnyv-rezultaty-doslidzhennya-znan-stavlennya-ta-povedinky-ukraintsiv-schodo-tuberkulozu#_ftnref1. Accessed 18 Sept 2020.

[29] World Health Organization. Companion handbook to the WHO guidelines for the programmatic management of drug-resistant tuberculosis. Geneva; 2014. https://apps.who.int/iris/bitstream/handle/10665/130918/9789241548809_eng.pdf?sequence=1. Accessed 19 August 2020. 25320836

[30] Sineke T, Evans D, Schnippel K, van Aswegen H, Berhanu R, Musakwa N (2019). The impact of adverse events on health-related quality of life among patients receiving treatment for drug-resistant tuberculosis in Johannesburg, South Africa. Health Qual Life Outcomes.

[31] Laxmeshwar C, Stewart AG, Dalal A, Kumar AMV, Kalaiselvi S, Das M (2019). Beyond ‘cure’ and ‘treatment success’: quality of life of patients with multidrug-resistant tuberculosis. Int J Tuberc Lung Dis..

[32] World Health Organization. A people-centred model of tuberculosis care. A blueprint for eastern European. Geneva; 2017. https://www.euro.who.int/__data/assets/pdf_file/0004/342373/TB_Content_WHO_PRO_eng_final.pdf. Accessed 19 August 2020.

[33] World Health Organization. WHO policy on collaborative TB / HIV activities Guidelines for national programmes and other stakeholders. Geneva; 2012. https://apps.who.int/iris/bitstream/handle/10665/44789/9789241503006_eng.pdf;jsessionid=68622ED295D634E6C4EEBEE9D08BD625?sequence=1. Accessed 19 August 2020. 23586124

[34] Seddon JA, Godfrey-Faussett P, Jacobs K, Ebrahim A, Hesseling AC, Schaaf HS (2012). Hearing loss in patients on treatment for drug-resistant tuberculosis. Eur Respir J.

[35] Pontali E, Sotgiu G, Tiberi S, D’Ambrosio L, Centis R, Migliori GB (2017). Cardiac safety of bedaquiline: a systematic and critical analysis of the evidence. Eur Respir J.

[36] Gupta R, Geiter LJ, Hafkin J, Wells CD (2015). Delamanid and QT prolongation in the treatment of multidrug-resistant tuberculosis. Int J Tuberc Lung Dis..

[37] Ferlazzo G, Mohr E, Laxmeshwar C (2018). Early safety and efficacy of the combination of bedaquiline and delamanid for the treatment of patients with drug-resistant tuberculosis in Armenia, India, and South Africa: a retrospective cohort study. Lancet Infect Dis.

[38] Alene KA, Clements ACA, McBryde ES, Jaramillo E, Lönnroth K, Shaweno D (2018). Mental health disorders, social stressors, and health-related quality of life in patients with multidrug-resistant tuberculosis: a systematic review and meta-analysis. J Infect.

[39] Penina O (2017). Alcohol-related causes of death and drinking patterns in Moldova as compared to Russia and Ukraine. Eur J Popul.

[40] Skiles MP, Curtis SL, Angeles G, Mullen S, Senik T (2018). Evaluating the impact of social support services on tuberculosis treatment default in Ukraine. PLoS One.

[41] de Vries G, Tsolova S, Anderson LF, Gebhard AC, Heldal E, Hollo V (2017). Health system factors influencing management of multidrug-resistant tuberculosis in four European Union countries - learning from country experiences. BMC Public Health.

[42] Harries AD, Dlodlo RA, Brigden G, Mortimer K, Jensen P, Fujiwara PI (2019). Should we consider a ‘ fourth 90 ’ for tuberculosis ?. Int J Tuberc Lung Dis..

[43] United Nations Development Program; Stop TB Partnership. The Legal Environment Assessment for TB in Ukraine. Kyiv; 2018. https://www.ua.undp.org/content/ukraine/en/home/library/democratic_governance/legal-environment-assessment-for-tuberculosis-in-Ukraine.html. Accessed 19 August 2020.

[44] United Nations Office for Project Services; Alliance for Public Health; Stop TB Partnership. Communities, rights and gender TB tools assessment Ukraine. Kyiv; 2018. http://aph.org.ua/en/news/final-study-report-within-communities-rights-and-gender-tb-tools-assessment/. Accessed 19 August 2020.

[45] USAID. Quality of tuberculosis services assessment: global tools - MEASURE evaluation. New York; 2020. https://www.measureevaluation.org/resources/publications/tl-19-41. Accessed 19 August 2020.

[46] Arsenault C, Roder-DeWan S, Kruk ME. Measuring and improving the quality of tuberculosis care: a framework and implications from the lancet Global Health Commission. J Clin Tuberc Other Mycobact Dis. 2019;16(1001122):1–6. 10.1016/j.jctube.2019.100112 PMC6716550 31497655

